# Influencing factors and application prospects of CO_2_ flooding in heterogeneous glutenite reservoirs

**DOI:** 10.1038/s41598-020-58792-z

**Published:** 2020-02-04

**Authors:** Jinkai Wang, Yuanpei Zhang, Jun Xie

**Affiliations:** 1 College of Earth Science and Engineering, Shandong University of Science and Technology, Qingdao, 266590 China; 2 Laboratory for Marine Mineral Resources, Qingdao National Laboratory for Marine Science and Technology, Qingdao, 266237 China

**Keywords:** Sedimentology, Climate and Earth system modelling

## Abstract

In recent years, large amounts of CO_2_ emissions have led to many environmental problems. Developing a reasonable approach to carbon dioxide emissions is one of the most important issues for the sustainable development of human civilization. Compared with CO_2_ geological storage, CO_2_ flooding has greater advantages and a higher utilization efficiency. CO_2_ flooding technology has been successfully applied to many types of reservoirs, such as conventional sandstone reservoirs, low-permeability reservoirs, and carbonates. In this paper, the feasibility of CO_2_ flooding in glutenite reservoirs is studied. First, the glutenite reservoir is divided into fine stages, and reasonable vertical development units are determined. On this basis, the distribution scale, genetic environment and formation mode of the glutenite reservoir are characterized in detail and their spatial distribution rules are depicted in three dimensions. Then, the influencing factors of CO_2_ flooding in the glutenite reservoir are analysed via reservoir numerical simulation technology. The effects of reservoir thickness, reservoir heterogeneity, macropores, dominant channels and fracturing on CO_2_ flooding efficiency are evaluated individually, and a set of reasonable parameters constituting an evaluation system for CO_2_ flooding in the glutenite reservoir is established. Finally, based on parameter optimization, the disadvantaged conditions of CO_2_ flooding in glutenite reservoirs are optimized, and their effects are gradually eliminated. In view of the characteristics of glutenite reservoirs, a unique three-dimensional well pattern arrangement is designed, different injection modes are optimized, and suitable injection agents are tested. These methods are conducive to improving the effect of reservoir parameters on CO_2_ flooding efficiency, allowing these reservoirs to be used for CO_2_ flooding. Based on these new methods, the displacement effect of the Yanjia-Yongan glutenite reservoir is predicted. Our results show that the reservoir has been developed efficiently and achieved a high recovery rate, the displacement front of the CO_2_ has become more uniform, and the sweep range has become wider. After extending the successful application of CO_2_ flooding in this reservoir to all of the glutenite reservoirs in the entirety of the Bohaiwan Basin, we predict that the oil recovery rate may reach 40%, with a cumulative oil recovery rate of approximately 3.04 × 10^8^ t and a total CO_2_ consumption of 1.672 × 10^8^ t. Thus, the proposed approach not only can improve the atmospheric environment but can also greatly improve the efficiency of oil displacement.

## Introduction

**Figure 1:**
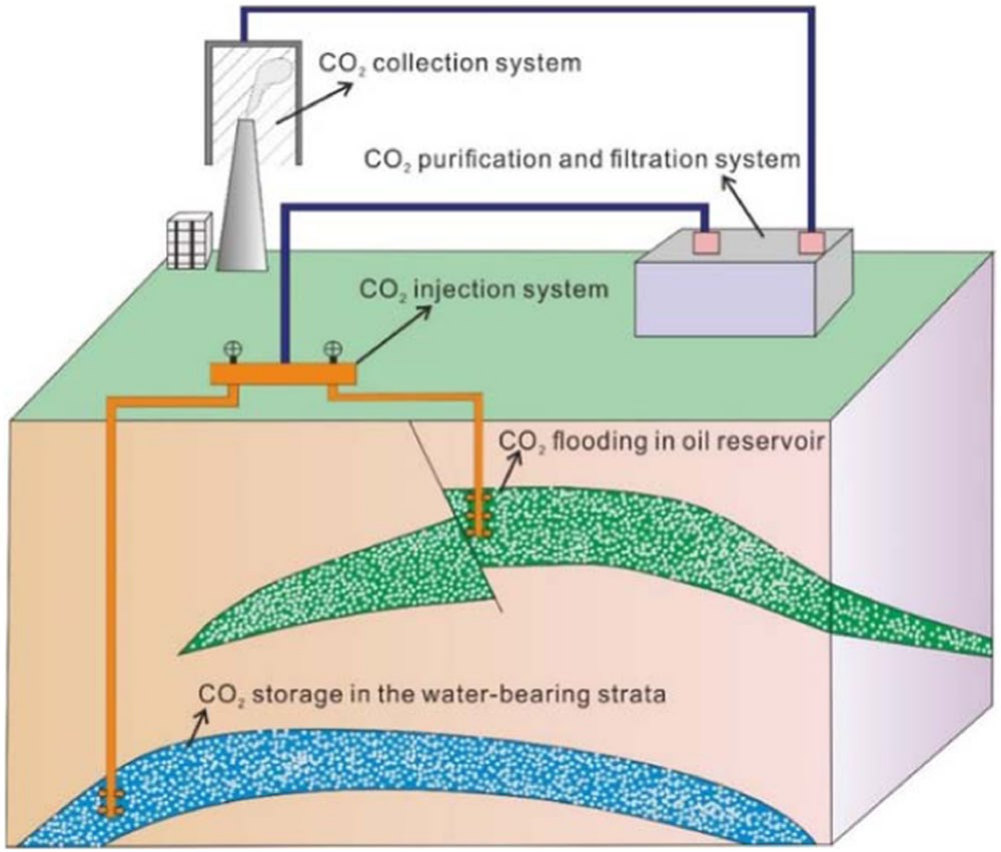
Current methods of CO_2_ utilization, collection and storage.

In recent years, human demand for fossil energy has increased, which has resulted in a rapid increase in the amount of greenhouse gases emitted into the atmosphere each year; this has had a great impact on the Earth’s environment. However, we cannot sacrifice economic development to reduce greenhouse gas emissions, and it is difficult to find a balance between these two objectives. Therefore, we must consider that large amounts of greenhouse gases have been discharged, are currently being discharged or will be discharged, and we must determine how to eliminate or minimize their harm to the environment. CO_2_ is the main component of greenhouse gases, and its content may be as high as 70%. Therefore, effective treatment of CO_2_ is the main way to reduce the harm of greenhouse gases. At present, there are two main ways to address CO_2_: one approach is geological storage, in which the collected CO_2_ is injected directly into an underground aquifer for storage, and the other is to use the aquifer for CO_2_ flooding in the oil development process. In comparing the economic and utilization ratios of the two methods, CO_2_ flooding is a two-pronged approach. At present, CO_2_ flooding technology is relatively mature and has been widely used around the world. Among countries that use the technique, the United States has the highest CO_2_ flooding efficiency, accounting for more than 80% of the world’s total CO_2_ displacement. However, the development of CO_2_ flooding technology in China is relatively lacking, and this method has not been widely used. Most of the Chinese reservoirs in which CO_2_ flooding has been implemented are homogeneous and low-permeability sandstone reservoirs, which are relatively simple^1–6^. In addition, studies have shown the feasibility of using CO_2_ flooding in shale oil reservoirs or in the carbonate reservoirs^7,8^. These studies have proven that CO_2_ flooding can produce higher oil displacement efficiency and has great application prospects (Fig. 1).

**Figure 2:**
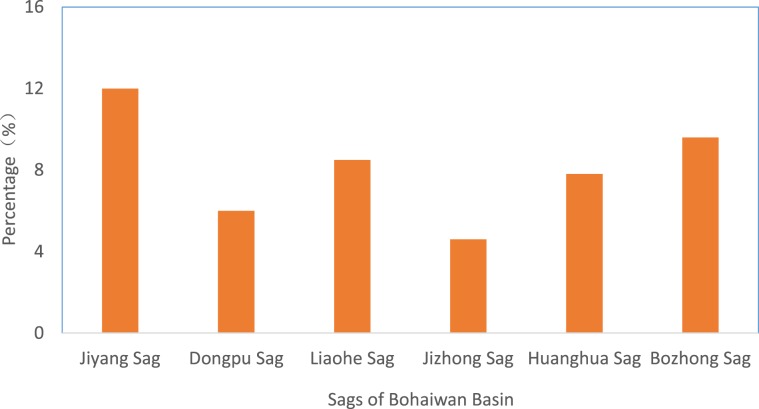
The proportion of glutenite reservoirs in the depressions of the Bohaiwan Basin.

Our literature review shows that the research on CO_2_ flooding mainly focuses on low-permeability reservoirs and seldom involves glutenite reservoirs. However, glutenite reservoirs are widely distributed in the filling period of fault basins in eastern China. These reservoirs are stacked deposits formed by gravity flow deposits such as alluvial fans, nearshore underwater goods, diluvial fans, deep-water turbidite fans and slump turbidite fans. They are generally distributed along the margin of continental lake basin steep slope zones and steep fault slopes, deep valleys and gully beam facies at the bottom of the fault basin. The glutenite reservoirs in the Bohaiwan Basin of China account for approximately 8% of the total oil reserves, and the reserves are massive^9^ (Fig. 2).

The burial depth of glutenite reservoirs is generally great, their permeability is low, and their oil-bearing types are complex; therefore, it is difficult to achieve an effective recovery via conventional production methods. For example, the Yanjia-Yongan glutenite reservoir of Dongying Sag’s recovery factor has been low due to conventional water flooding and cannot achieve satisfactory results. Since improving oil recovery via conventional development methods is difficult, new methods should be employed to develop this kind of reservoir. Using the successful development experience of other types of reservoirs for reference, we speculate that this kind of reservoir is better suited for carrying out CO_2_ flooding and can achieve better recovery. However, to achieve effective CO_2_ flooding in this kind of reservoir, many related technologies must be studied. For example, screening criteria, including the basic geological parameters of the genesis, the morphological characteristics and distribution patterns of glutenite, the influence of pore structure and heterogeneity characteristics of glutenite on CO_2_ flooding, and the types of well patterns, injection modes and injection types of CO_2_ flooding, all need to be further studied to provide a feasible scheme for carrying out CO_2_ flooding development in the glutenite reservoir.

Gravity flow-deposited conglomerate reservoirs are quite different from other types of sandstone reservoirs (fluvial facies and delta facies). This difference is not only reflected in their external factors, such as shape, scale and distribution law, but also in their internal micro-pore structure, which leads to great differences in the seepage mechanism and distribution pattern of fluids in reservoirs. Therefore, studying this type of reservoir in detail is necessary to develop carbon dioxide flooding in a glutenite reservoir. In studying the sedimentary facies and sedimentary model of glutenite, many scholars have carried out systematic research with the help of a large amount of logging data (such as resistivity scanning imaging logging data), analysis of laboratory data and coring well data^10^. These studies are mostly based on core analysis and summarize the characteristics of different lithologies, sedimentary structures and rhythms of glutenite. Thus, they establish the relationship between rock and logging, and these studies systematically investigate the sedimentary characteristics, genetic types and sedimentary evolution model of glutenite by combining rock and electricity. The sedimentary facies types of the glutenite body determined in these studies are mainly the offshore subaqueous fan, sublacustrine fan and fan delta^11,12^. Researchers believe that the main controlling factors of the formation of glutenite are the intensity of fault activity, the change of lake level and the supply capacity of provenance, and the different distributions of glutenite are caused by the influence of factors in different regions^13^.

Generally, the sedimentation of the glutenite body is mainly an event deposit; its sedimentation rate is fast, its thickness is large, and the internal structure is complex. Its vertical lithological difference is not obvious, and the lateral distribution is unstable, which makes it difficult to distinguish and compare the sedimentary cycles, and it is difficult to characterize the glutenite body accurately. In view of these realities, researchers have implemented many innovations in classifying glutenite stages, as follows: using the astro-stratigraphic climate cycle theory driven by the astronomical period to control the isochronism of glutenite in the time domain, then determining the stratigraphic division scheme^14^; using FMI (Formation MicroScanner Image) data to establish a stratification model and dividing the lithologic assemblage of glutenite to finely divide the sedimentary stages^15^; using high-order spectrum and time-frequency analysis technology to achieve the division of single well stages to determine the large-scale sedimentary stages of glutenite cross-correlation tracking^16,17^; and using the INPEFA (Integrated Prediction Error Filter Analysis) cycle analysis technique to characterize the interior of the sandy conglomerate and to divide the channels of different stages and levels, which allows the realization of the division of the sedimentary stages^18^. Similarly, many technical methods can be used for reference to characterize the internal structure of glutenite. For example, the multi-attribute fusion technology system based on seismic imaging (seismic multi-attribute analysis technology, constrained seismic inversion technology) can realize the quantitative identification of complex glutenite and its internal fracture prediction^19–21^. The study of glutenite reservoir quality differences based on laboratory data can quantitatively characterize the heterogeneity of the glutenite at different stages and further characterize the different facies belts, diagenetic products and distribution characteristics of glutenite at different diagenetic stages; this approach can also determine the influence of these parameters on reservoir quality^22–24^. However, how to develop the glutenite is still an open question. At present, most of the glutenite reservoirs in the oilfield are exploited as unconventional reservoirs (such as low-permeability reservoirs, fractured reservoirs, etc.), focusing on the impact of the fracture system on development. Before being developed, the reservoir is first fractured artificially, then the conventional water flooding is used for overall development^25,26^. Horizontal well development is also an effective method in glutenite reservoirs with a large thickness and few vertical interbeds. In addition, some shallow heavy oil reservoirs are developed by thermal recovery technology^27^.

CO_2_ flooding technology has been widely used in reservoir development and has achieved effective results. Because of the relatively high cost of implementing this technology, most of its applications are in low-permeability reservoirs and unconventional reservoirs. In addition, conventional reservoirs may adopt CO_2_ flooding at the high water cut stage in later stages of development^28^. According to different reservoir properties, many researchers have designed a variety of CO_2_ flooding simulation experimental devices to study the mechanisms underlying changes in the physical properties of reservoirs that are caused by CO_2_ flooding and to simulate the effect of CO_2_ flooding under physical conditions. Researchers have obtained the key parameters of the range of miscible displacement by using these experiments^29–33^. In addition to physical experiments, many component models used for CO_2_ flooding have been established, and numerical simulation technology has also been widely used in the evaluation and prediction of the effects of CO_2_ flooding. By establishing the two-dimensional plane model and three-dimensional model and using computers to simulate the displacement process, the efficiency of CO_2_ flooding and its influencing factors under different conditions can be evaluated, which can provide reasonable guidance for the implementation of carbon dioxide flooding^34–37^. The heterogeneity has a great influence on the EOR (Enhanced oil recovery) effect of CO_2_ flooding, the fluid distribution in the process of oil displacement has a good correlation with the permeability of rocks. Generally speaking, with the increase of the degree of heterogeneity of rocks, the oil recovery of the reservoir decreases significantly^38^, even for the reservoir with weak heterogeneity, the recovery of immiscible and miscible flooding will be greatly reduced^39^. Because the greater the permeability difference (the stronger the heterogeneity) is, the greater the displacement heterogeneity caused by CO_2_ fingering is, the more serious the unidirectional breakthrough of fluid is, and the recovery of reservoir will be greatly affected^40^. A large number of physical simulation experiments show that in the conventional sandstone reservoir, water gas alternate displacement is more suitable for CO_2_ flooding than continuous displacement. The enhanced oil recovery of WAG flooding may be due to the dominant viscous force, the difference in gravity will increase the sweep efficiency of injected fluid, and the capillary action will start to take effect, which can greatly improve the oil recovery^41^. The experimental results also confirmed that wag displacement performed better in all cases and reached the highest recovery rate^42^.

In addition, in the process of CO_2_ flooding, much waste gas will be produced, which can affect the safety and efficiency of CO_2_ flooding. In the treatment of these produced gases, new technical methods, such as the collection method, treatment process and reinjection scheme of the produced gas, have also been researched and have been proven to improve the efficiency of carbon dioxide flooding^43,44^. On this basis, researchers have determined the reservoir parameters suitable for CO_2_ flooding, and they have established a reservoir evaluation system that includes technical screening, economic screening, feasibility fine evaluation and the recommendation of the optimal gas injection block for CO_2_ flooding^45^. While the development of these technologies provides a sound reference for CO_2_ flooding, none of these studies focused specifically on glutenite reservoirs. Therefore, unique techniques have been implemented to establish a CO_2_ flooding evaluation scheme suitable for glutenite reservoirs.

## Research Ideas and Methods

**Figure 3:**
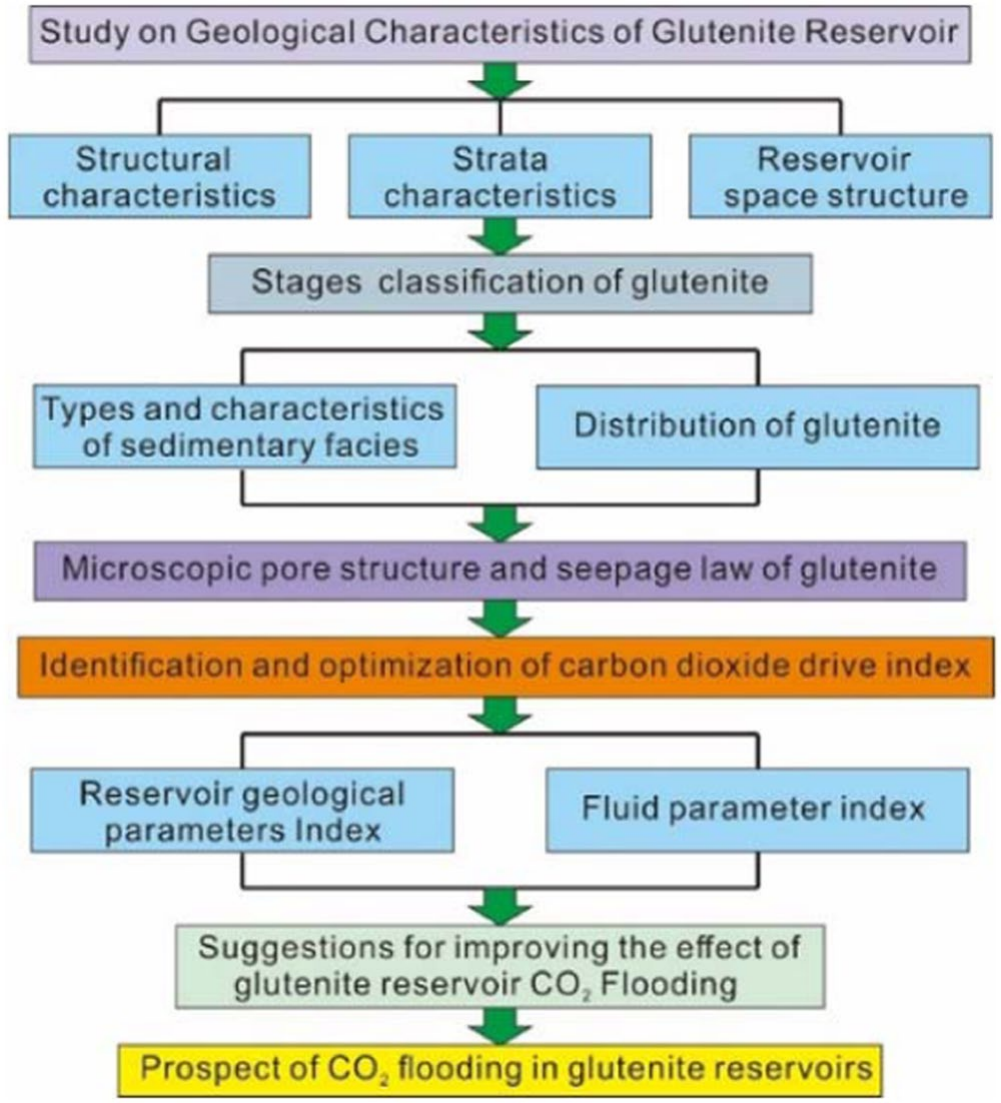
Research Ideas and Procedures.

Glutenite reservoirs represent the key direction for unconventional oil and gas exploration, as they occupy a large proportion of China’s oilfields, and glutenite reservoirs are especially prevalent in fault basins in eastern China. For example, in Dongying Sag of the Bohaiwan Basin, this type of reservoir accounts for approximately 15% of the total oil reserves and is a key exploration and development area. Therefore, updating the technology used in the development of glutenite reservoirs and improving their development level would be of great help in enhancing the future recovery of the old oilfields in eastern China. In this paper, the Yanjia-Yongan glutenite reservoir in Dongying Sag is taken as an example and the technology of enhancing oil recovery by CO_2_ flooding in glutenite reservoirs is systematically studied by using physical experiments and numerical simulation methods (Fig. 3):

1. Study the structural evolution patterns and sequence structure characteristics of the reservoir and divide the sedimentary stages of the glutenite body.
2. Analyse the rock characteristics and sedimentary facies characteristics of the glutenite body and clarify its sedimentary type and distribution patterns.
3. Analyse the physical characteristics and micro-pore structure characteristics of the glutenite reservoir and clarify the fluid seepage pattern.
4. Optimize the reasonable range of each parameter according to the standard of CO_2_ flooding.
5. Analyse the influencing factors of carbon dioxide displacement effect in glutenite reservoirs from the aspects of sedimentation and diagenesis.
6. Suggest improvements for the CO_2_ displacement effect of glutenite reservoirs; these suggestions are posited and optimized.
7. Predict the economic benefits and prospects of CO_2_ flooding in glutenite reservoirs in the Bohaiwan Basin.

## General Situation of the Research Area

**Figure 4:**
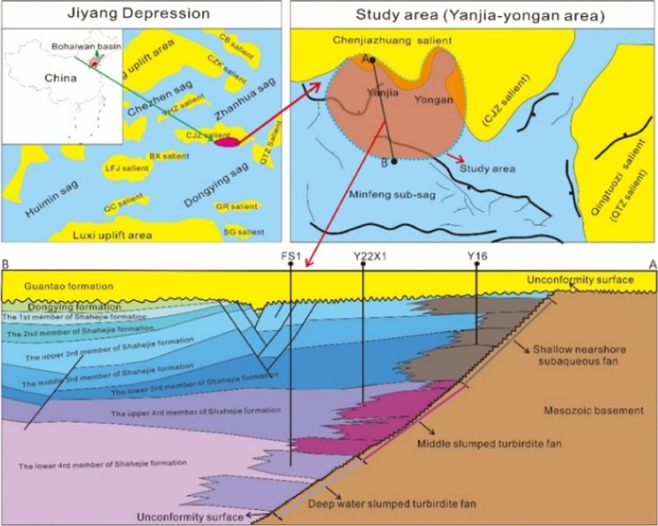
Tectonic location map and stratigraphic characteristics of the study area.

The Yanjia-Yongan glutenite reservoir is located in Kenli County, Dongying City, Shandong Province. Its tectonic location is on the north side of the central anticline of the Dongying Sag, Jiyang Depression of the Bohaiwan Basin in the eastern segment of Tuozhuang-Shenglicun-Yonganzhen fault zone. Its northern boundary is the Chenjiazhuang uplift, and the eastern boundary is the Qingtuozi uplift; these two uplifts are paleo-uplifts of granitic gneiss. Its southern area is the Minfeng oil-generating depression. The study area is within the overlap zone of the paleo-fault-denudation surface on the southern flank of the Chenjiazhuang uplift. Its structural characteristics are mainly controlled by the bedrock palaeogeomorphology and the activity of the Chennan fault on the southern edge of the uplift. There are fewer faults in the sedimentary area of the glutenite fan, and the structure is relatively simple; the overall tectonic framework is characterized by high mountains, steep slopes and alternating gullies and berms (Fig. 4).

## Characteristic Description of the Glutenite Bodies

### Characteristics of the stratigraphic sequence

**Figure 5:**
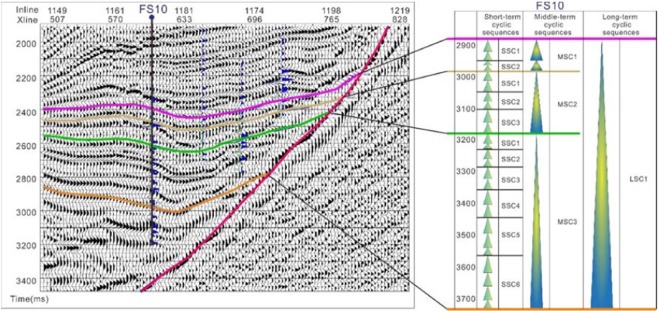
Characteristic description of Yanjia-Yongan glutenite bodies.

In the lower part of the upper ES_4_ member, the glutenite bodies are very thick and are formed by the overlapping of offshore subaqueous fan channels, and there is very little mudstone in this region. Up to the upper part of ES_4_, the mudstone layer thickness increases and the outer fan subfacies of the gully deep water fan begins to appear. Except for the late stage of the upper part of ES_4_, there is no large-scale lake flooding sequence; therefore, the whole upper part of ES_4_ is defined as a long-term cycle of stage III. The sequence division of the single well shows that the upper part of ES_4_ can be divided into three stage IV medium-term base level cycles (MSC1, MSC2, MSC3) that correspond to three sand formation groups. Furthermore, MSC3 can be divided into 6 short-term base-level cycles, MSC2 into 3 short-term base-level cycles and MSC1 into 2 short-term base-level cycles (Fig. 5).

### Petrological characteristics

**Figure 6:**
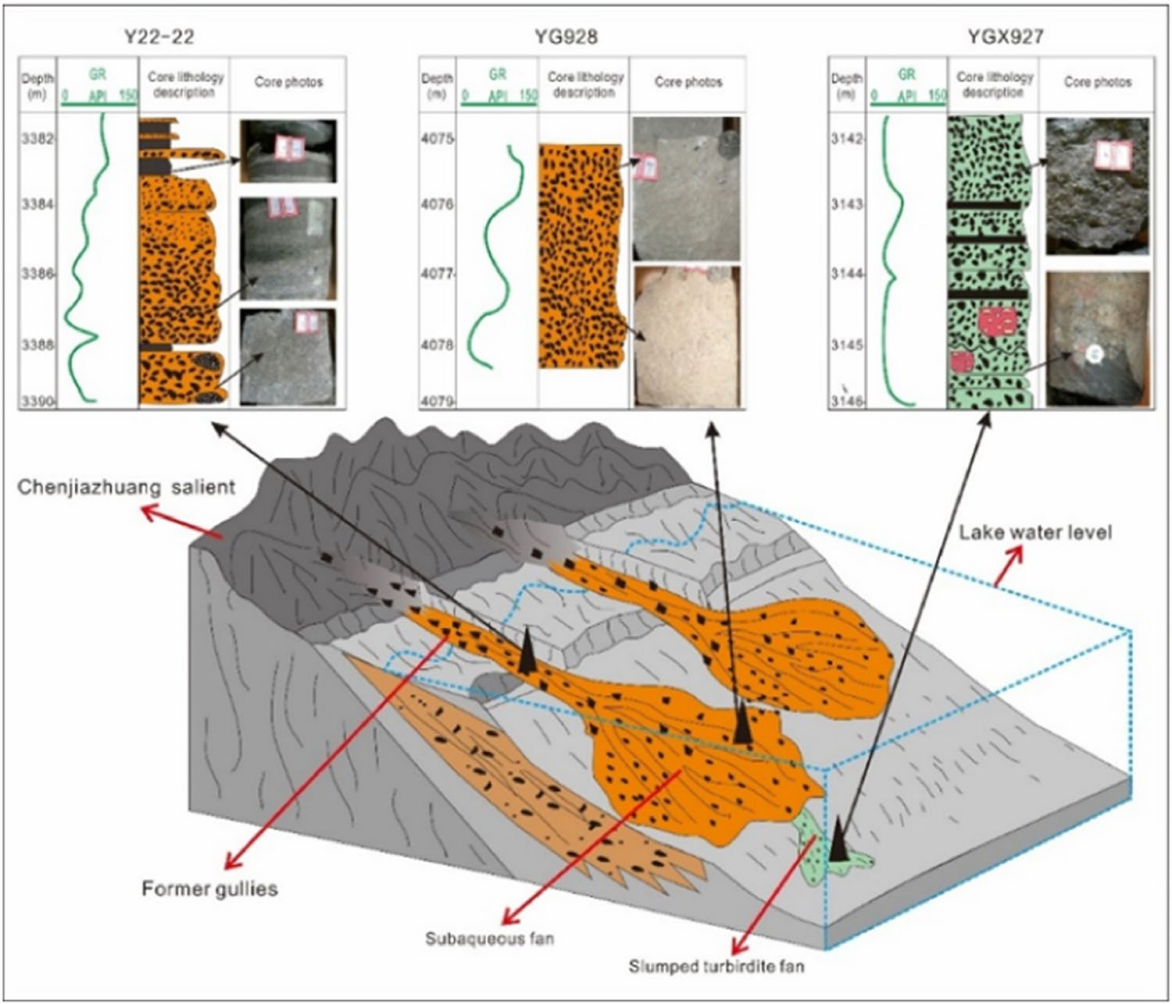
Sedimentary facies model of the Yanjia-Yongan area.

The upper part of the ES_4_ deposition period represents an obvious retrogradation process. Large quantities of debris are transported by floods through ancient gullies, and gravity flow depositional systems are formed in deep water areas. However, from the characteristics of the sediments, the grain size, structure, sedimentary structure and mudstone content of upper and lower sediments are obviously different. The sedimentary environment and ancient landform analysis show that the water body expanded and the paleo-gully was submerged, which resulted in a change of the sedimentary environment in the late stage of the upper part of ES_4_. A comprehensive analysis shows that the main facies in the study area are offshore underwater fans, gully deep water fans, slump turbidite fans and gravity channels. In the lower part, the offshore underwater fan depositional system is dominant; in the upper part, the gully deep fan depositional system is the main type (Fig. 6).

### Physical characteristics

**Figure 7:**
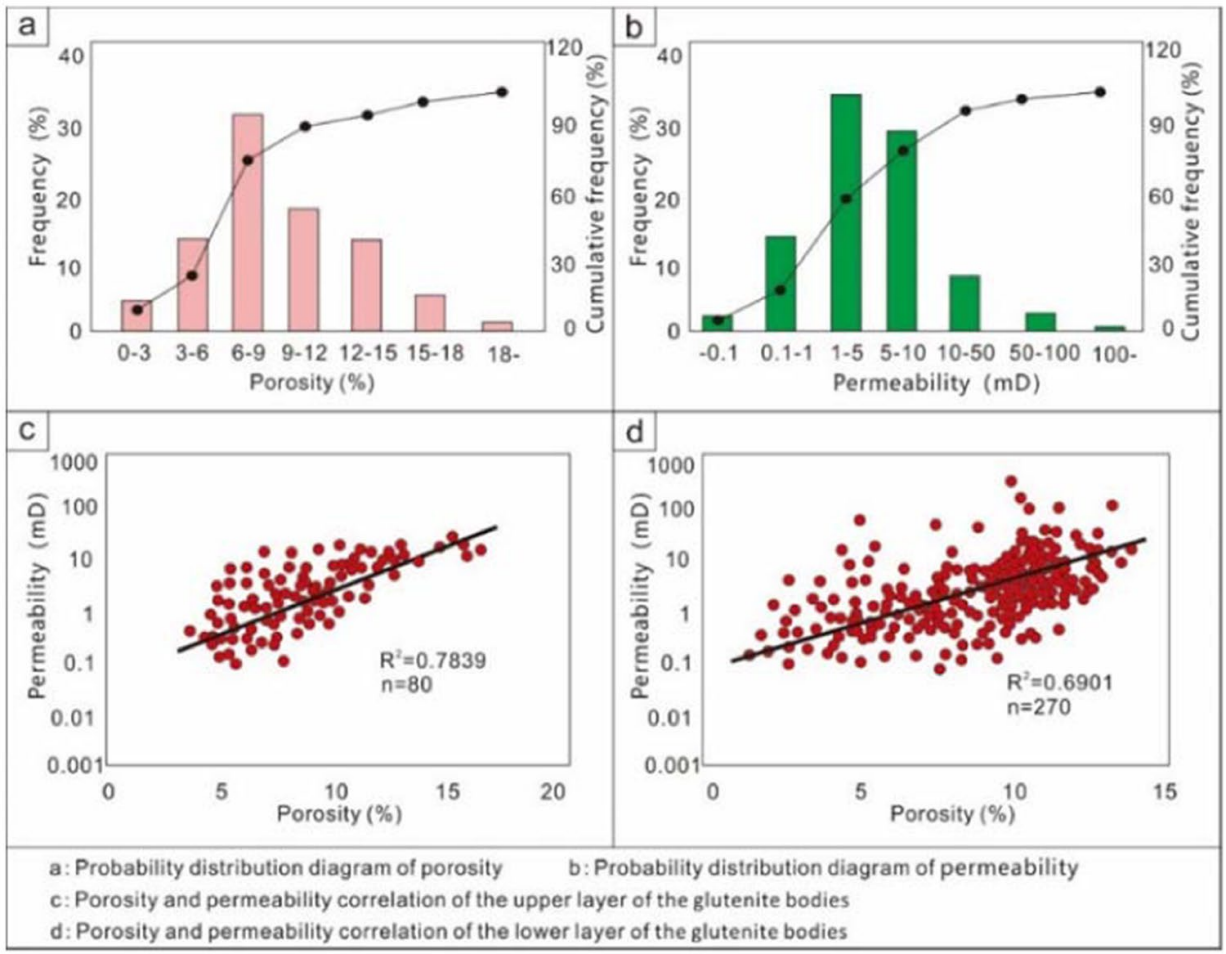
Statistical map of rock physical properties in the Yanjia-Yongan area.

The porosity of the glutenite reservoir is mainly between 5% and 10%, and the permeability is mainly between 1 and 10 mD. It is a typical low-porosity and low-permeability reservoir. The porosity and permeability are positively correlated (Fig. 7).

**Figure 8:**
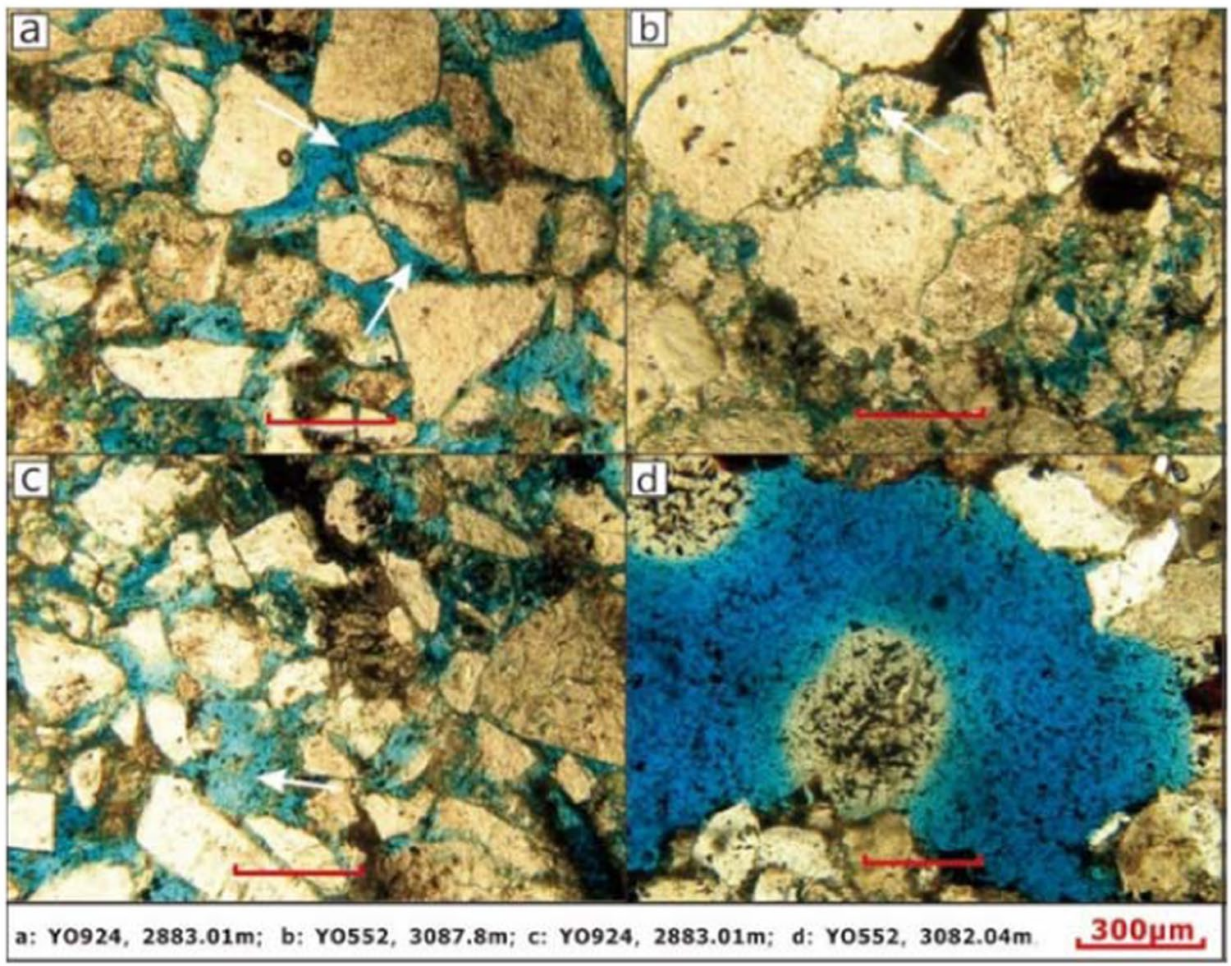
Pore characteristics of rocks.

Intergranular pores are the most important pore type in the Yanjia-Yongan glutenite bodies, which mainly include residual primary pores and secondary pores. Secondary intergranular pores are mainly formed by the dissolution of carbonate cements and the clay matrix, and some debris grain margins can also be dissolved to form secondary pores. The number of intragranular pores is also relatively large, and the most common is the intragranular pore formed by feldspar dissolution, followed by debris. In addition, there are a few fractures and extra-large pores in the Yanjia-Yongan glutenite bodies (Fig. 8).

**Figure 9:**
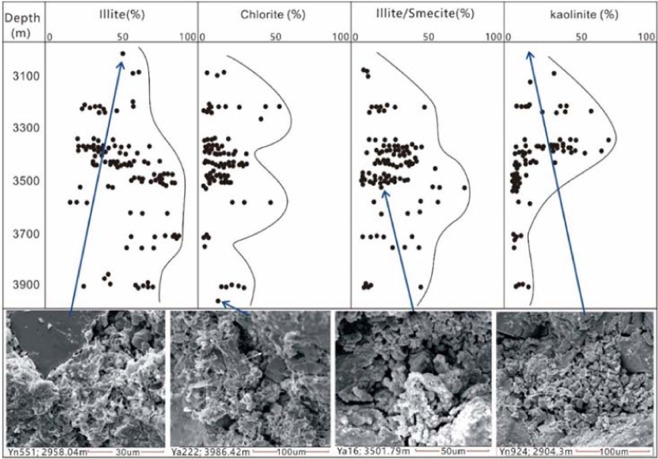
Statistics of clay mineral contents.

Clay minerals are abundant in sandy conglomerate reservoirs in the Yanjia-Yongan area. According to an X-ray diffraction analysis of whole rock minerals, the clay mineral content in the Yanjia area ranges from 1% to 33%, with an average content of 5.98%. The clay mineral content in the Yongan area ranges from 3% to 35%, with an average of 10.55%. X-ray diffraction analysis of the clay minerals shows that kaolinite, chlorite, illite, palygorskite and other clay minerals are widely distributed in the reservoirs. Illite is the most abundant clay mineral, with an average relative content of 58.24% in the Yanjia area and 45.06% in the Yongan area. In the pores, the shape of illite is mainly flaky or filamentous, while some illite shows hair-like filaments and forms illite membranes on the surface of particles (Fig. 9).

There are so many clay minerals in glutenite that they can easily block the pore throat of the rock, which has a substantial destructive effect on the permeability of sandstone. For the CO_2_ flooding reservoirs, if there is a large amount of chlorite in rocks, it will aggravate the acidity effect of the reservoir and greatly damage the pore.

## Feasibility evaluation of CO_2_ flooding

### Feasibility of geological parameters and fluid conditions

CO_2_ flooding greatly aids in the development of low-permeability reservoirs, but it requires more rigorous geological conditions and reservoir fluid properties, including reservoir depth, formation pressure, saturation pressure, reservoir temperature, reservoir physical properties, crude oil viscosity, gas-oil ratio and other parameters; these properties must meet certain conditions to carry out CO_2_ flooding and achieve the best development effect. CO_2_ flooding experiments have been successfully carried out in many reservoirs in China, and most of them have achieved effective results, such as in the Jilin Oilfield, Daqing Oilfield, Shengli Oilfield, Changqing Oilfield, etc. These successful instances provide helpful references for this study. In this paper, we compare the parameters of the glutenite reservoir in the study area with the successful cases (Table 1). Most of its basic parameters meet the requirements of CO_2_ flooding. The unfavourable factors, such as heterogeneity, fracture, gas cap, edge and bottom water, are also within a relatively reasonable range. Therefore, CO_2_ flooding can theoretically be carried out in the Yanjia-Yongan glutenite reservoir.

**Table 1:** Feasibility comparison table for carbon dioxide flooding.

Evaluating indicator	Data range	Reservoir characteristics	Evaluation result
Reservoir thickness	35–80	Average 60 m	Not suitable
Connectivity of oily regions	Medium to good	Good	Suitable
Reservoir abundance	120–210 × 10^4^ t/km^2^	More than 100 × 10^4^ t/km^2^	Suitable
Reservoir depth	2800–3800 m	Average 3500 m	Suitable
Reservoir heterogeneity	Medium to stronger	Stronger	Common
Fracture development degree of reservoir	/	A few	Suitable
Reservoir porosity	5.5–12.6%	Average 9.6%	Suitable
Reservoir permeability	3.58–19.66 × 10^–3^ μm^2^	Average 7.92 × 10^–3^ μm^2^	Suitable
Formation pressure	28.5–37.9 MPa	Average 34.3 MPa	Suitable
Reservoir temperature	107–114 °C	Average 110 °C	Suitable
Crude oil density (reservoir condition)	0.6308–0.6929 g/cm^3^	Average 0.6623 g/cm^3^	Common
Viscosity of crude oil (reservoir condition)	1.08–4.43 mPa.s	Average 2.49 mPa.s	Suitable
Dissolved gas-oil ratio	115.2–145.3 m^3^/m^3^	125.7 m^3^/m^3^	Suitable
Reservoir structure	/	Weak edge and bottom water, no gas cap	Suitable

### Minimum miscible pressure (MMP) evaluation of miscible flooding

The successful implementation of carbon dioxide flooding shows that to achieve a beneficial displacement effect, the injected carbon dioxide must be miscible with the underground crude oil. Miscible flooding is a tertiary oil recovery technology and is an important method used to improve oil recovery. Its mechanism is to make the displacement agent and crude oil miscible in the formation, causing the interface between them to disappear. At this time, the capillary force in porous media decreases greatly and crude oil flows out more easily, which can greatly improve oil recovery. For CO_2_ flooding, to make CO_2_ and crude oil miscible in the formation, the most important parameter is the formation pressure; that is, the reservoir pressure must be at least able to achieve the minimum miscibility pressure (MMP). The MMP is related to many factors, such as the composition of crude oil, formation temperature, formation pressure, etc., but it is the composition of crude oil that plays the main role. Table 2 is the basic composition of crude oil.

**Table 2:** The basic composition of crude oil.

Component	CO_2_	N_2_	C_1_	C_2_	C_3_	iC_4_	NC_4_	iC_5_	NC_5_
Percentage content (%)	0.453	0.301	28.411	1.007	1.003	0.455	0.368	1.366	2.563
Component	C_6_	C_7_	C_8_	C_9_	C_10_	C_11_	C_12_	C_13_	C_14+_
Percentage content (%)	1.878	4.115	6.321	7.013	5.501	4.621	3.579	3.518	27.527

Thus, we must determine an optimal method to obtain the MMP of the reservoir. These methods include the analogy method, physical experiment method, empirical formula method, numerical simulation method, flash simulation method, etc. In this paper, we use these methods to calculate the MMP of the Yanjia-Yongan glutenite reservoir (Table 3). The calculation results in the table show that the average MMP is approximately 29.38 MPa, which is much lower than the original reservoir pressure (the original reservoir pressure of Yanjia-Yongan glutenite reservoir is 34.3 MPa). Therefore, this reservoir is theoretically suitable for CO_2_ miscible flooding.

**Table 3:** MMP calculation results of CO_2_ flooding in the Yanjia-Yongan glutenite reservoir.

MMP calculation method		Calculation formula	Formula analysis	MMP (MPa)
Empirical formula	Y-M correlation^57^	${\rm{MMP}}=1.5832+0.19038{\rm{T}}-0.00031986{{\rm{T}}}^{2}$	T: temperature, K	28.52
	J-P correlation^58^	MMP = Pci + 0.00703a(T-Tci) + 0.00703I(βM-Mi)^2^	M: Oil molecular weight Mi: Gas molecular weight Tci: Gas critical temperature, K Pci: Gas critical pressure, MPa I = 1.2762; α = 18.9; β = 0.285	27.46
	NPC correlation^59^	Comparison with reasonable scope	ρ > 0.893, MMP = 28.12 0.893 > ρ > 0.876, MMP = 21.09 ρ < 0.876, MMP = 7.03	29.15
	Improved PR equation of state correlation^60^	MMP = 0.052 × 10^2.772-(1579/RT)^ R = 1.8 T + 492	T: temperature, K	28.56
Numerical simulation	The model of glutenite body is used to add various practical parameters of the reservoir for numerical simulation, the method is Peng-Robinson EOS.			29.23
Flash simulation experiment	MMP simulation was carried out by flash experiment			29.75
Analogy method	By comparing the characteristics of this reservoir with other reservoirs that have already carried out CO_2_ displacement.			30.15

The MMP of Yanjia-Yongan glutenite reservoir is 4 MPa lower than the original formation pressure, so in the original state (not put into development), the reservoir can directly carry out miscible flooding. Even after a short period of development (the recovery interval is less than 10%), the conditions of CO_2_ miscible flooding are easy to achieve. Therefore, the numerical simulation in this paper is based on miscible flooding. It is speculated that for general sandstone reservoir, CO_2_ miscible flooding can increase oil recovery by 15%, compared with non miscible flooding.

## Result

Theoretically, the third oil recovery method of carbon dioxide flooding can be used in glutenite reservoir. However, because of its particularity, it is necessary to analyze the influence of different parameters (reservoir thickness, reservoir heterogeneity, macropores and fissures) on displacement efficiency, so as to maximize the role of carbon dioxide flooding and produce more remaining oil.

### Reservoir numerical simulation model

#### Geological model

**Figure 10:**
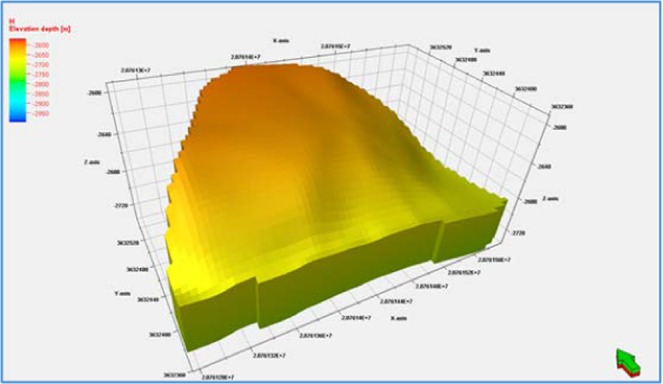
3D geological model of the glutenite body.

Based on the conclusion of fine geological study of glutenite, a three-dimensional geological model is established. The model is built in the form of corner grid, its three-dimensional grid system is 64 × 40 × 20, and the total grid number is 52000. The grid steps in direction I and j are 5 m (Fig. 10). Sedimentary facies model is completed by deterministic modeling method. Physical property model (porosity model and permeability model) is calculated by stochastic interpolation method under the control of microfacies model.

#### Geological model

**Figure 11:**
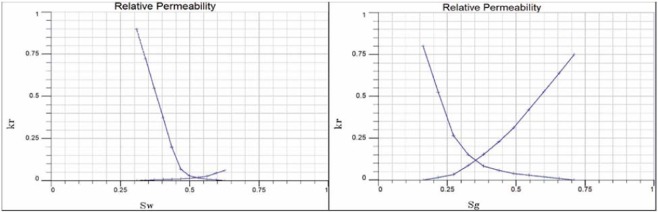
Relative permeability curve of the glutenite body.

After the static 3D geological model is established, the fluid parameters (pressure, temperature, relative permeability, etc.) need to be assigned to complete the initialization of the reservoir model. According to the input balance data table (reference pressure, reference depth or reference grid), as well as the porosity, permeability, saturation and other data of each small layer, the simulator uses the depth average balance initialization method to initialize the reservoir, and calculates the discrete interpolation to each grid. The reservoir pressure and temperature in the numerical simulation model are assigned according to the original values in Table 1, which are consistent with the actual reservoir parameters. The original relative permeability curve of controlling reservoir seepage is shown in Fig. 11, which is adjusted properly in the process of numerical simulation to make the result closer to the reality.

#### Mathematical model fluid seepage

According to the conservation of mass and Darcy’s law^46^, the differential equation of fluid seepage can be obtained as follow:

**Differential equation of water phase seepage:**

$$\nabla \cdot [\frac{{K}_{rw}}{{\mu }_{w}}{\rho }_{w}K\nabla {P}_{w}]+\frac{{q}_{w}}{{V}_{b}}=\frac{\partial }{\partial t}[\varphi {\rho }_{w}{S}_{w}]$$

**Seepage equation of oil and gas components:**

$$\nabla \cdot [\frac{{K}_{ro}}{{\mu }_{o}}{\rho }_{o}{F}_{n}K\nabla {P}_{o}+\frac{{K}_{rg}}{{\mu }_{g}}{\rho }_{g}{G}_{n}K\nabla {P}_{g}]+\frac{{q}_{n}}{{V}_{b}}=\frac{\partial }{\partial t}[\varphi ({\rho }_{o}{S}_{o}+{\rho }_{g}{S}_{g}){Z}_{n}]$$

**Auxiliary equation:**

$$1={S}_{w}+{S}_{o}+{S}_{g}$$

$${P}_{g}={P}_{o}+{P}_{cog}$$

$${P}_{w}={P}_{o}+{P}_{cow}$$

**Boundary conditions:**

Closed outer boundary $\nabla P|{\Gamma }_{out}=0$

**Mathematical model solution:**

Pressure difference equation

$$\begin{array}{c}\Delta [\theta {(\frac{{K}_{rw}}{{\mu }_{w}}{\rho }_{w}K)}^{m}\Delta ({{P}_{o}}^{m+1}-{{P}_{cwo}}^{m}-{\gamma }_{w}^{m}D)+{(\frac{{K}_{ro}}{{\mu }_{o}}{\rho }_{o}K)}^{m}\Delta ({{P}_{o}}^{m+1}-{\gamma }_{o}^{m}D)\\ \,+{(\frac{{K}_{rg}}{{\mu }_{g}}{\rho }_{g}K)}^{m}\Delta ({{P}_{o}}^{m+1}-{{P}_{cwg}}^{m}-{\gamma }_{g}^{m}D)]\\ \,+\theta \cdot {q}_{w}^{m+1}+{(\mathop{\sum }\limits_{i=1}^{{N}_{c}}{q}_{m})}^{m+1}\frac{{V}_{b}}{\Delta t}({\varphi }^{m+1}{\alpha }^{m+1}-{\varphi }^{m}{\alpha }^{m})=0\end{array}$$

Saturation difference equation

$${{\rm{S}}}_{w}^{m+1}=\frac{\Delta \Delta {P}_{w}^{m+1}+{q}_{w}^{m}+\frac{Vb}{\Delta t{\varphi }^{m}{\rho }_{w}^{m}{S}_{w}^{m}}}{\frac{Vb}{\Delta t{\varphi }^{m+1}{\rho }_{w}^{m+1}}}$$

Meaning of letters in the formula:

n – Quantity of oil and gas components; K – Permeability, 10^–3^ μm^2^; ϕ – Porosity; S – Saturation;

K_r_ – Relative permeability; P – Pressure, MPa; ρ – Mole density, mol/cm^3^; μ – Viscosity, mPa·s;

q – Production rate, mol/s; F_n_ – Fluid componentdecimal; G_n_ – Gas componentdecimal;

Z_n_ – Total component, decimal; o, g, w – Oil gas and water phase; V_b_ – Unit volume, cm^3^;

P_c_ – Capillary pressure, MPa; Г – Model boundary; θ –Volume ratio of hydrocarbon to water

### Influence of reservoir thickness

**Figure 12:**
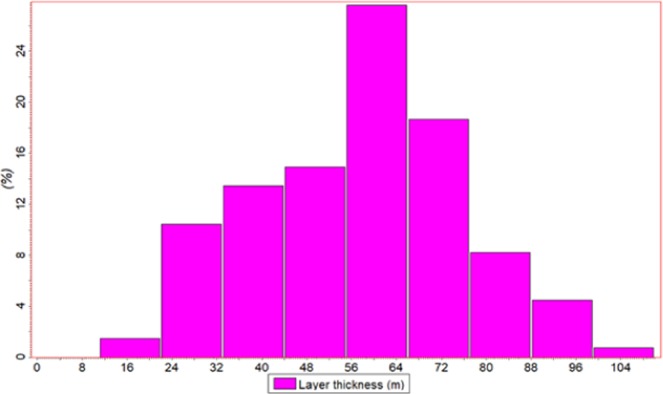
Thickness statistical map of each sublayer of the Yanjia-Yongan glutenite body.

The sedimentary facies in the study area is a nearshore subaqueous fan, and the sedimentary system is complex and lacking in fossil biomarkers and stable traceable mudstone barriers. Therefore, it is difficult to divide the glutenite body. Previous researchers classified the glutenite body as the middle cycle level, with the average thickness of each layer exceeding 200 m^47,48^. However, it is impossible to carry out CO_2_ flooding-related research with such a thick development layer. In this paper, many new techniques and methods have been applied to the stratification study, such as the sedimentary inversion method, Michaelis cycle method, Morlet continuous wavelet transform method, multi-mineral model analysis method, Fischer graphic method, numerical simulation method of sedimentary dynamics, etc^49^. Based on these methods, we have found many stratification marks in addition to mudstones. The Yanjia-Yongan glutenite body has been subdivided into 12 sublayers, each with an average thickness of 60 m and a distribution range of 10–100 m, which greatly increases the feasibility of CO_2_ flooding. Figure 12 is a statistical map of the thickness of each sublayer after the subdivision of the Yanjia-Yongan glutenite body.

**Figure 13:**
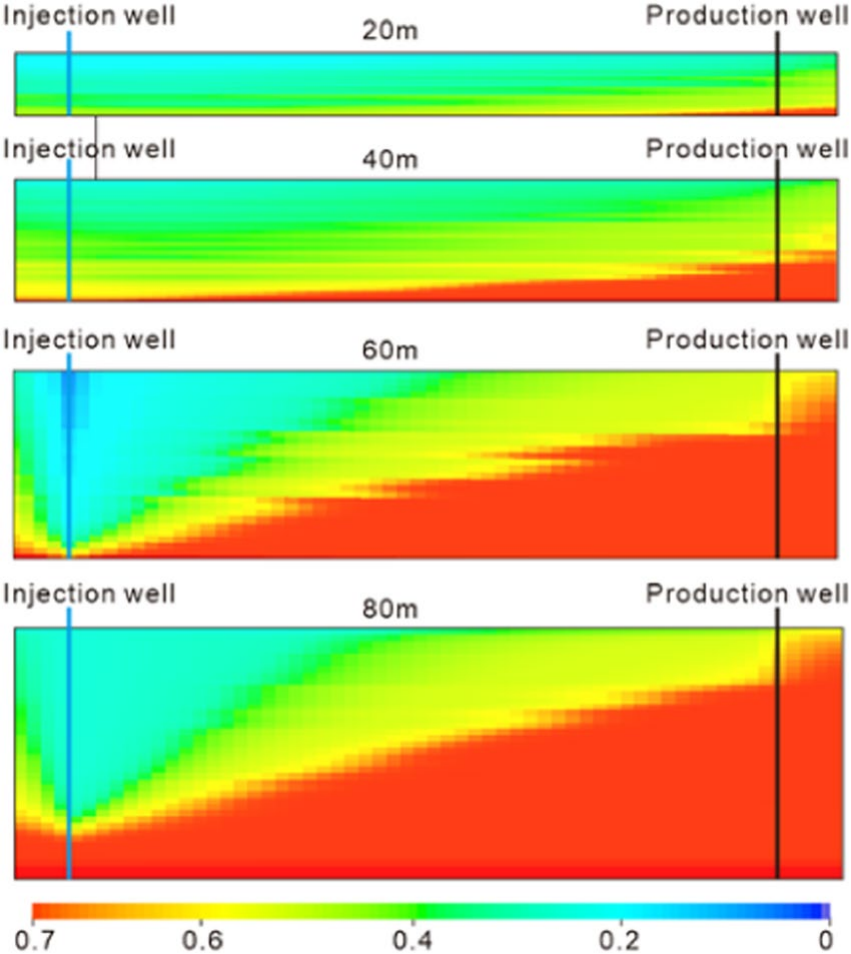
Comparison of the carbon dioxide flooding effect in reservoirs with different thicknesses.

According to the adaptability evaluation criterion of the CO_2_ flooding reservoir^50,51^, the single layer thickness of the Yanjia-Yongan glutenite reservoir exceeds 60 m, which is not suitable for CO_2_ flooding in theory. The problem is that if CO_2_ is injected into such a thick reservoir, the gas is prone to gravity overlap, forming injection-production channels in the upper part of the reservoir; the lower part of the reservoir also becomes less effective, thereby resulting in a large amount of remaining oil. Figure 13 is an oil displacement effect diagram of CO_2_ flooding in different reservoirs of different thicknesses. It shows that with an increase in reservoir thickness, oil displacement efficiency decreases gradually. Due to the influence of gas buoyancy, this subregion is difficult to reach, especially in the lower part of the reservoir near the production wells. Once the injection-production channel is connected at the top, the lower part of the reservoir will no longer be used, and the remaining oil cannot be exploited at the top of the reservoir.

### Influence of reservoir heterogeneity

**Figure 14:**
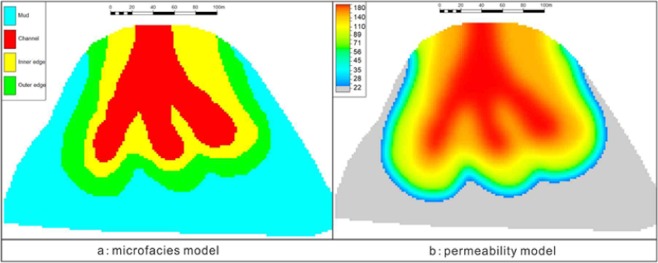
Sedimentary microfacies model and permeability model of the Yanjia-Yongan glutenite reservoir. (**a**) the microfacies model was established by using deterministic modeling simulator, the property values can be assigned directly from existing data. First the planar attribute with microfacies code is established according to its planar distribution map, and then the modeling process of microfacies is constrained according to the attribute plane. (**b**) the modeling method of permeability is mainly stochastic interpolation under the control of microfacies model. Random simulation is carried out in the boundary of microfacies, each one has a unique limit, which ensures the matching of sedimentary law and physical conditions.

The reservoir heterogeneity is also an important factor affecting the CO_2_ flooding effect. If the reservoir heterogeneity is weak and the lateral variation of physical properties is relatively stable, the CO_2_ displacement front will be relatively uniform, the diffusion range will be wide, and the oil displacement efficiency will be higher. Conversely, if the reservoir heterogeneity is strong, a gas inrush belt is easily formed, which is not conducive to gas diffusion; thus, the oil displacement efficiency of the reservoir will be lower^52,53^. Generally, the reservoir heterogeneity is greatly influenced by the sedimentary facies, which controls the distribution of physical properties. However, the glutenite body belongs to the turbidity current sedimentary system, the boundaries between its microfacies are not obvious, and the reservoir physical properties are quite different around the whole glutenite body. The physical conditions of the sand body in the middle fan channel are relatively robust, the physical properties of sand body in the periphery are poor, and the overall physical properties of the sand body have obvious strip characteristics (Fig. 14).

**Figure 15:**
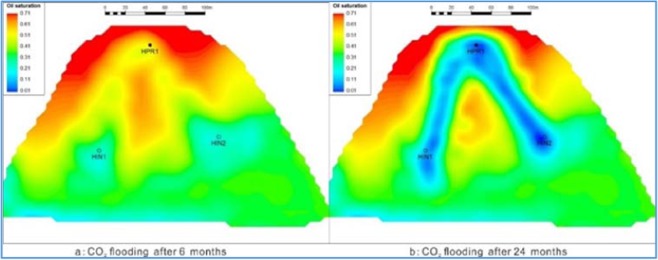
Fluid variation law of carbon dioxide flooding.

To characterize the effect of reservoir heterogeneity on CO_2_ oil removal, the corresponding mathematical model was designed and the numerical simulation model of CO_2_ flooding was established. Using this model to simulate the process of carbon dioxide flooding in glutenite can adequately reflect the degree of fluid affected by physical properties and sedimentary facies. Figure 15 is a carbon dioxide displacement effect plan of the Yanjia-Yongan glutenite reservoir (oil saturation variation pattern). The graph shows that the seepage of carbon dioxide has an obvious directivity in highly heterogeneous reservoirs. In the connection between injection wells and production wells, carbon dioxide rapidly advances along the high-permeability channel sand body, resulting in obvious high-permeability channels. With the increase in injection time, the continuous injection of carbon dioxide does not penetrate in other directions; instead, it mainly travels along the high-permeability belt of the channel sand body, and both the productivity of oil wells and the displacement effect gradually deteriorate.

### Influences of macropores and fissures

**Figure 16:**
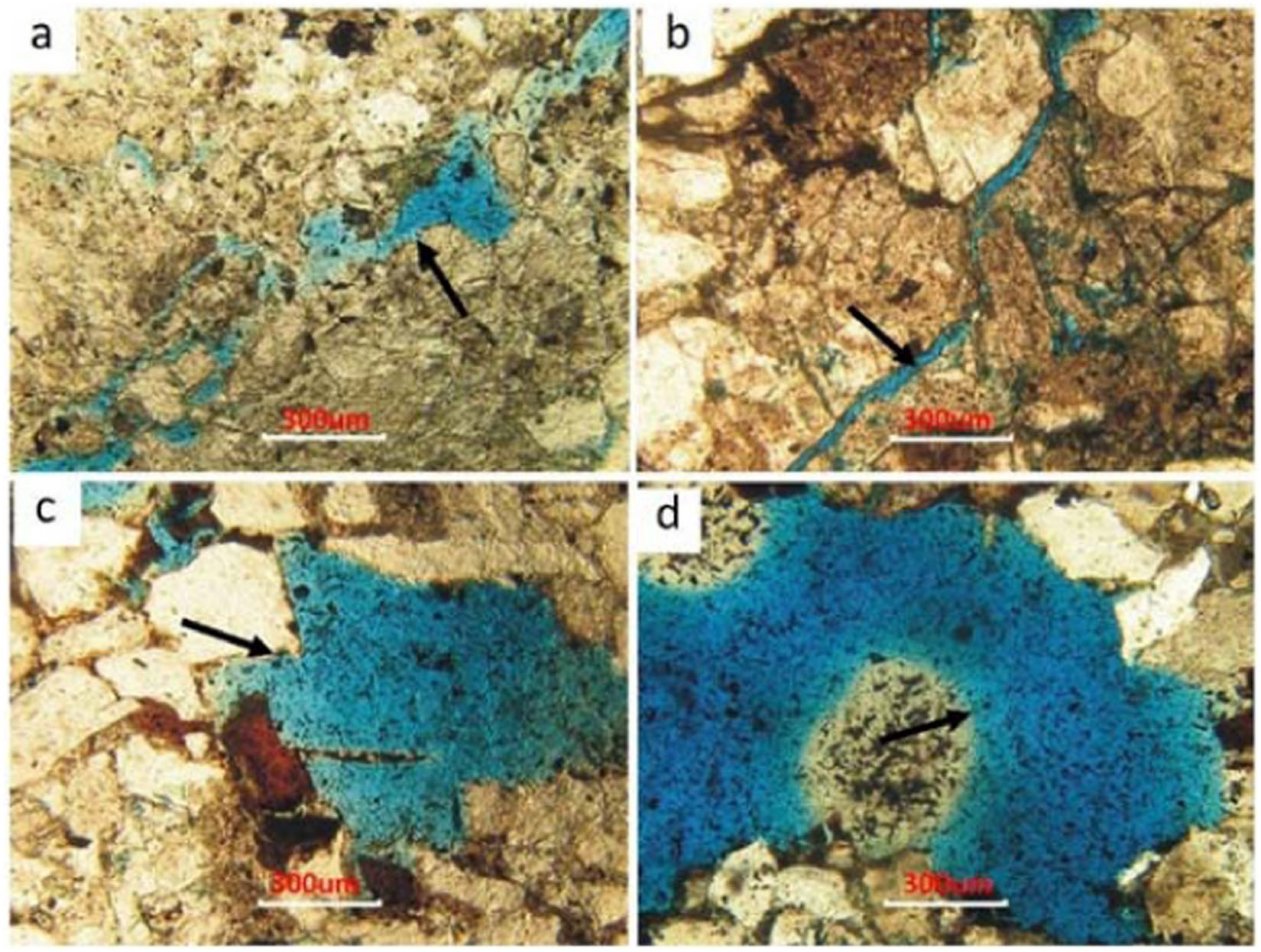
Structural fracture and extra-large pore characteristics of the Yanjia-Yongan glutenite body. (**a**) YO551, 3046.84 m; (**b**) YA16, 2019.09 m; (**c**) YO552, 3082.04 m; (**d**) YO552, 3082.04 m.

The lithology of the Yanjia-Yongan glutenite is mainly conglomerate, gravel sandstone and conglomerate-bearing sandstone. Because of its large burial depth and steep slope, its rocks are strongly reformed in the later stages, including via compaction, dissolution, tectonic deformation and so on. There are two main results of diagenesis. One result is compaction and tectonic compression, which leads to rigid particle compression (such as quartz and feldspar). When the overburden pressure exceeds the compressive strength of the particles, the particles rupture along their weak surface, resulting in tectonic fissures (Fig. 16a,b). Another result of diagenesis is dissolution; the dissolution solvents in the study area are mainly deep organic acids produced during hydrocarbon generation. The secondary pore development zones of the Yanjia-Yongan glutenite body are from 3280 m to 3580 m and from 3760 m to 3990 m, respectively. The main oil generation window in the study area is 2900 m to 4800 m, which is consistent with the depth of the secondary pore development zone. Therefore, we infer that these dissolution reagents mainly come from the hydrocarbon generation process. Calcite and feldspar are the main dissolution minerals in the study area. The maximum width of the cracks produced by dissolution can exceed 500 µm, which can be called giant cracks, resulting in fluid seepage into the pipe flow (Fig. 16c,d).

**Figure 17:**
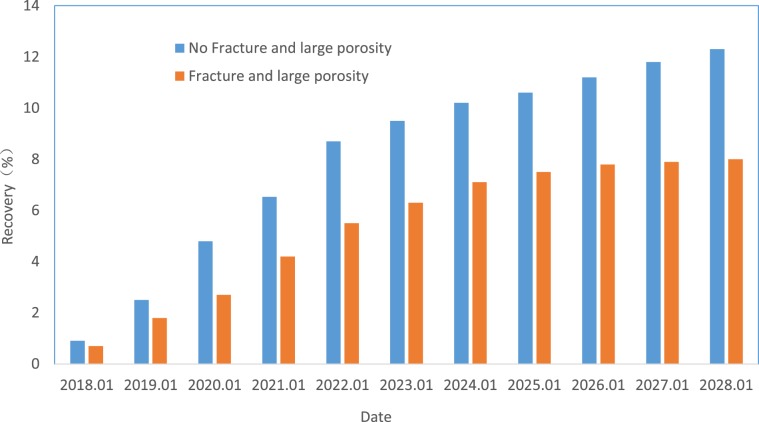
Comparison of CO_2_ flooding efficiency between the homogeneous and fracture models.

The existence of macropores and fractures in the sandy conglomerate reservoir will affect the percolation of CO_2_ and the displacement effect. Because the relative permeability of gas is much greater than that of oil, once a crack is encountered, it will rapidly burst, thereby seriously affecting the sweep efficiency in other directions. To determine the influence degree of fractures and macropores on CO_2_ flooding, we designed two models to carry out the numerical simulation: one model is the homogeneous model, which lacks fractures, and the other is the heterogeneous model, which includes natural fractures and macropores. These models can be used to compare the differences in CO_2_ flooding efficiency. Due to the tectonic movement and dissolution, there will be large pores and wide throat, which will change the seepage law of the fluid in the crude oil reservoir. The injected CO_2_ will quickly advance along these channels with good permeability, while the gas sweep efficiency in other parts will be greatly reduced, leading to the overall low recovery.Comparing the final recovery of the two models, the final recovery of the model with macropores and fractures is 40% lower than that of the homogeneous model (Fig. 17). Therefore, to achieve a better recovery rate, we should avoid cracks and pore development intervals during gas injection and choose relatively homogeneous areas as much as possible to carry out CO_2_ flooding.

**Figure 18:**
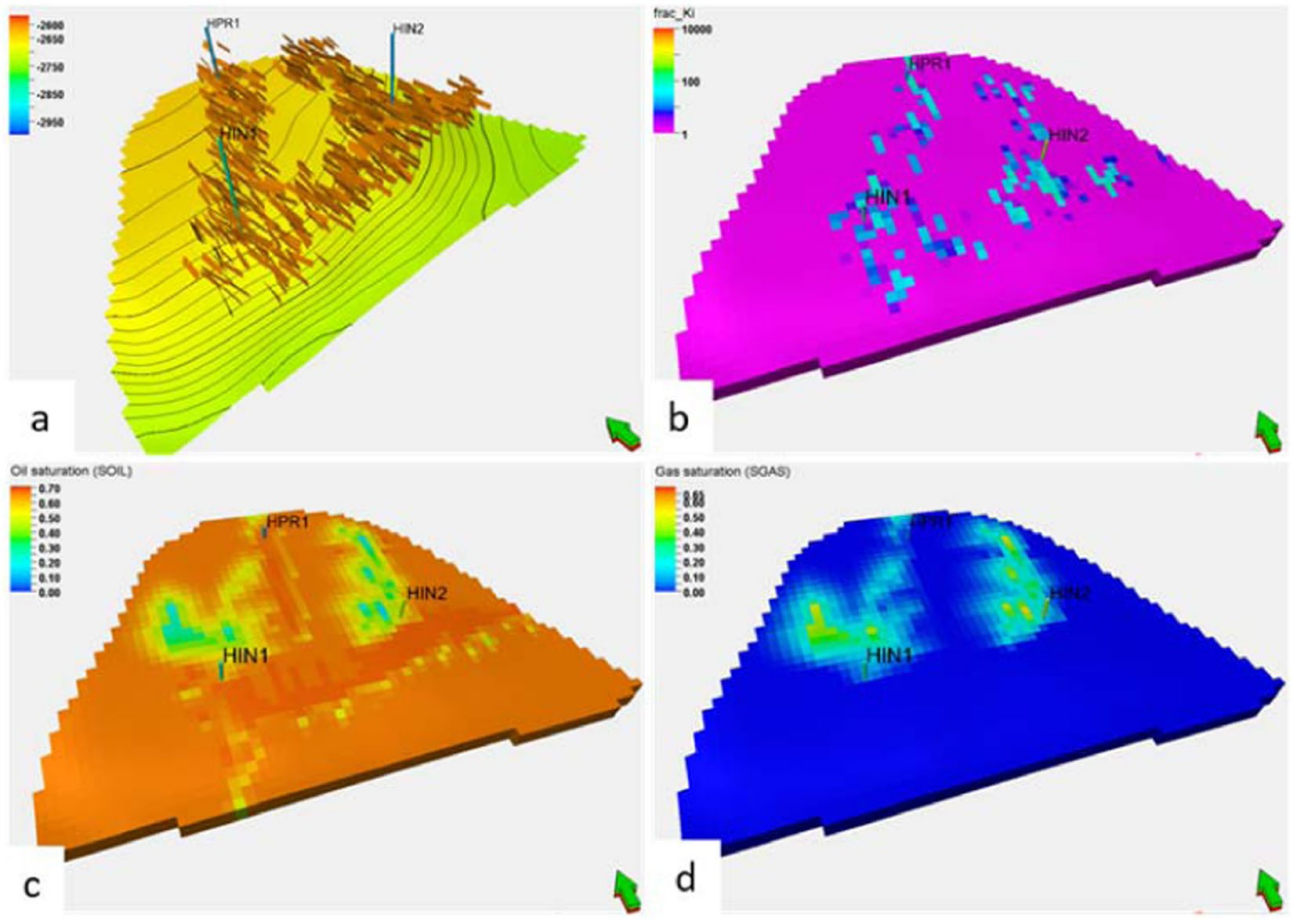
The effects of fractures and macropores on CO_2_ flooding. (**a**) Fracture model; (**b**) Physical property model; (**c**) Distribution of oil in the process of CO_2_ flooding; (**d**) CO_2_ concentration distribution in the process of CO_2_ flooding.

Figure 18 shows the effect of CO_2_ flooding in the glutenite reservoir with fractures and large pores. The figure shows that in the area with large pores and fractures, the concentration of CO_2_ obviously increases, and the oil displacement effect is relatively strong; in the area without fractures and with large pores, the oil displacement effect is poor. The establishment of an effective connection between the injection and production wells will have a great impact on the effect of CO_2_ flooding in the whole reservoir. The CO_2_ will only rush forward into the high-permeability channel, and other directions will no longer produce diffusion; not only does this not improve oil recovery over ordinary water flooding but it also causes a serious waste of resources.

## Discussion

### Condition improvement of CO_2_ flooding in a glutenite reservoir

The Yanjia-Yongan glutenite reservoir is characterized by high reservoir heterogeneity and poor physical properties; if only the traditional water flooding method is used to develop it, the effect on production will be very poor. Many glutenite reservoirs in Dongying Sag are in this situation; especially in the later stages of water injection, the water cut increases greatly and is difficult to control. The efficiency of oil wells becomes obviously decreased, and the reservoir recovery rarely exceeds 10%. At present, CO_2_ flooding is a relatively feasible method to improve oil recovery. However, we found that although the Yanjia-Yongan glutenite reservoir is theoretically suitable for CO_2_ flooding, some reservoir parameters do not meet the requirements of efficient CO_2_ flooding, such as the fracture development degree and reservoir thickness. We must therefore determine how to optimize the mode of CO_2_ flooding to achieve the highest oil displacement efficiency in glutenite reservoirs. According to the geological characteristics of the Yanjia-Yongan glutenite reservoir and the numerical simulation method, some suggestions are posited as follows:

#### How to solve the problems caused by reservoir thickness

**Figure 19:**
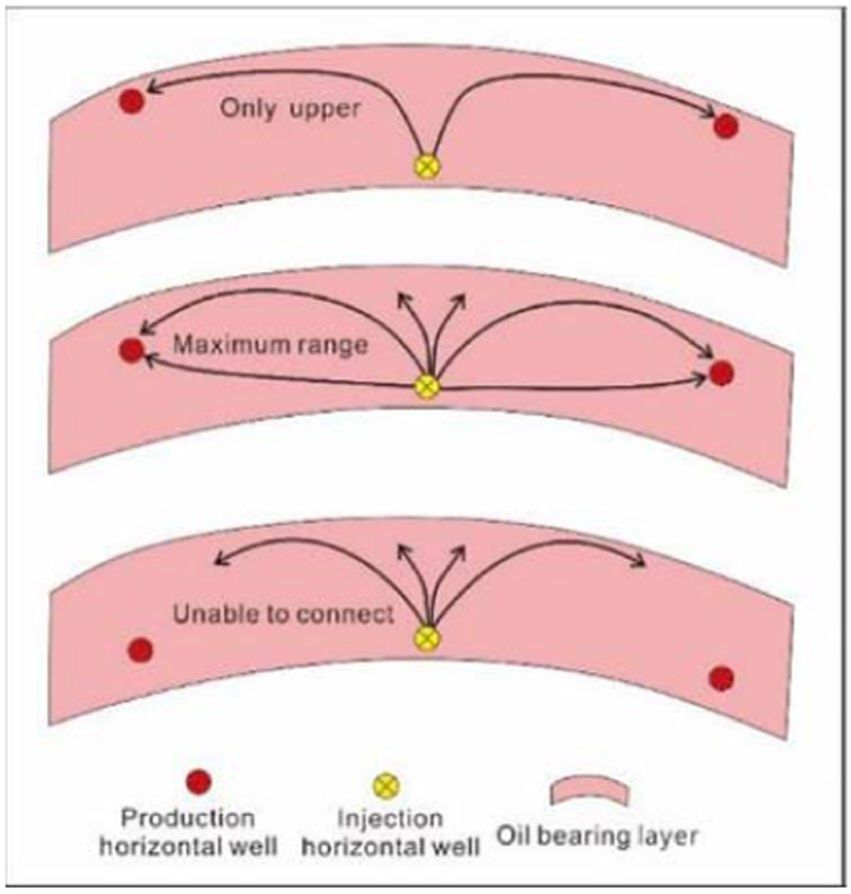
The corresponding relationship between horizontal wellbore location and displacement efficiency.

The single-layer thickness of the Yanjia-Yongan glutenite reservoir is greater than 60 m. There are many drawbacks to the use of a conventional vertical well pattern for CO_2_ flooding; for example, due to the influence of the gas buoyancy effect, the vertical oil displacement is not complete, and much residual oil will be retained at the bottom that cannot be recovered. In view of this reality, we have designed a three-dimensional horizontal well pattern to carry out CO_2_ flooding in the thick glutenite reservoir to address the effect of fluid gravity. This well pattern can make full use of the characteristics of the large thickness of the glutenite reservoir or can greatly offset the impact of the large thickness. The horizontal wells are designed at different depths of the reservoir to form a three-dimensional well pattern so that much of the residual oil caused by the gravity effect of the fluid can be effectively exploited (Fig. 19).

Influenced by bulk buoyancy, the injected CO_2_ will primarily displace the oil at the top of the reservoir; therefore, the basic principle of this three-dimensional well pattern is to locate the injection well at the bottom of the reservoir and the production well at the top of the reservoir. Generally, the injection wells can be appropriately close to the bottom boundary of the sand body, and the distance between them is relatively free and not strictly limited. Meanwhile, the location of the production wells should be strictly limited because their location directly affects the displacement efficiency. We must then determine the appropriate location of the oil well in the reservoir. We can identify this location according to Fig. 19, which shows that when the production wells are near the top of the reservoir (Fig. 19a), it is easy to establish a connection between the injection wells and production wells. Once this connection is successfully established, the lower part of the reservoir will not be displaced effectively, and most of the oil here will become residual oil. When the production wells are at the bottom of the reservoir (Fig. 19c), it is difficult to establish effective communication between the injection and production wells due to the gas buoyancy, which will lead to the worst possible displacement effect. When the gas is injected for a certain period, the pressure of the injection well will increase dramatically; thus, the CO_2_ flooding cannot continue. Therefore, our comprehensive analysis shows that the location of production wells should be located in the upper middle part of the reservoir (Fig. 19b). In this case, the process of carbon dioxide displacement can be divided into two stages: first, displacement begins at the top of the reservoir. When the crude oil, whose viscosity is reduced by CO_2_, seeps into the upper part of the production wellbore, it seeps downward and enters the production wellbore due to the influence of the pressure funnel and gravity near the wellbore. Then, the middle and lower parts of the reservoir begin to be completely displaced. With the continuous top displacement, the relationship between the injection wells and production wells is gradually established, and most areas between them will be displaced by CO_2_ under the combined action of gravity and buoyancy.

**Figure 20:**
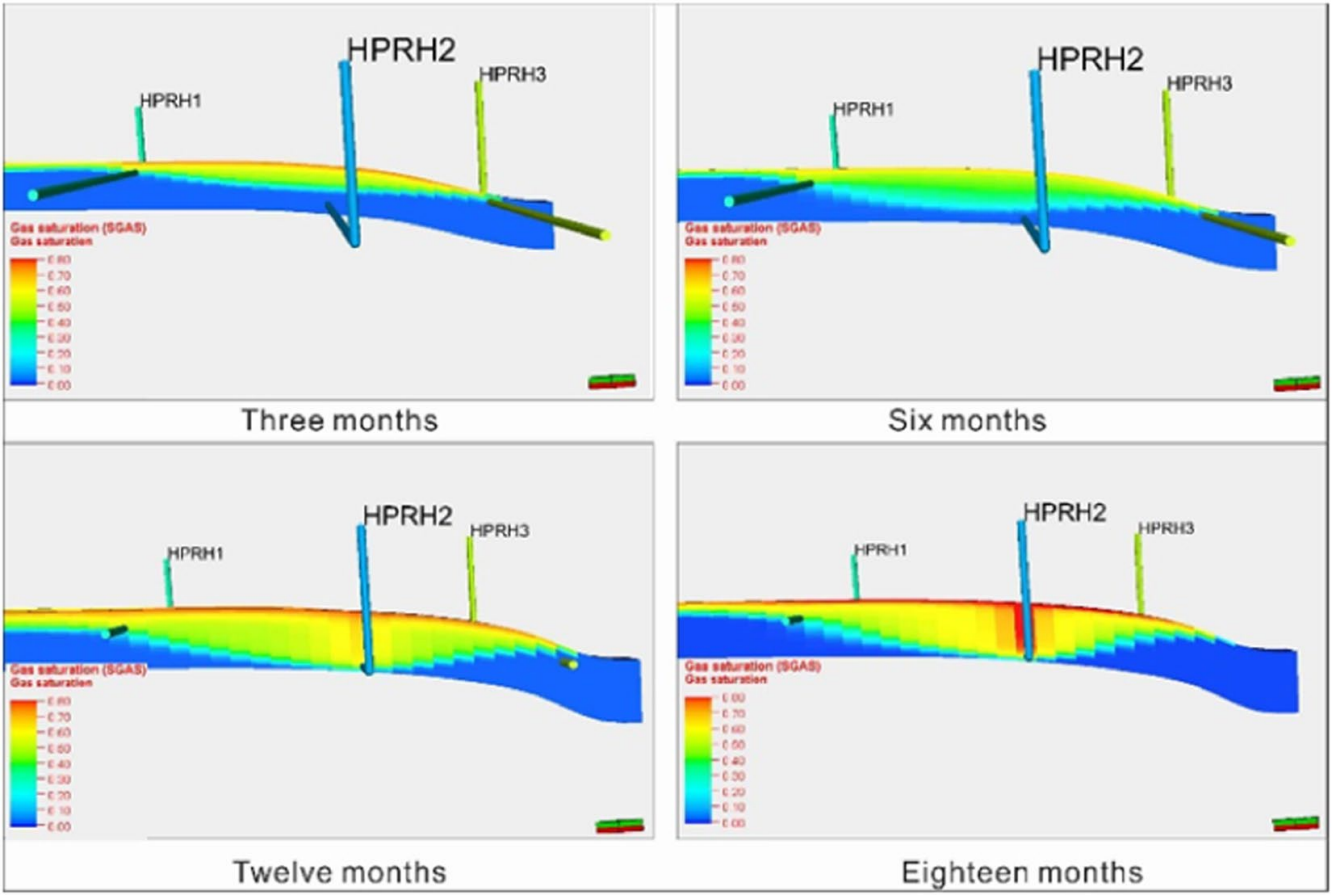
CO_2_ displacement effect map by using a three-dimensional horizontal well pattern.

Figure 20 is a prediction chart depicting the displacement effect of CO_2_ flooding using a 3D horizontal well pattern. The figure shows that in the initial stage of gas injection, low-viscosity fluids rapidly rise to the top of the reservoir and then push towards both sides. When they reach the upper part of the production well, they begin to move slowly downward. After approximately 3 months, the displacement front can reach the production horizontal wellbore. With the continuous increase in gas injection, the lower part of the reservoir is gradually displaced, and the final sweep efficiency of gas can exceed 80%.

#### Accounting for the influences of fracture sand macropores

Fracture systems commonly developed in glutenite reservoirs are the greatest obstacle to improving the efficiency of CO_2_ flooding because the fractures will become the dominant channels of small-molecular gas, which will make gas rapidly rush forward. Once the gas is connected between the injection and production wells, the displacement efficiency of CO_2_ will be greatly reduced (Fig. 16). Therefore, to obtain a higher oil displacement efficiency in such reservoirs, special treatments for large pores and fractures are needed to limit their conductivity, thus expanding the sweep range of CO_2_ and enhancing oil recovery. In theory, the diameter of CO_2_ molecules is approximately 3 A (0.3 × 10^–9^ m), and the diameter of water molecules is approximately 4 A (0.4 × 10^–9^ m). The conventional fracture width is (10–50) × 10^–6^ m, and the macropore width can reach 1000 × 10^–6^ m, which is much larger than its counterparts^54^. Therefore, if there are fractures or large pores in the reservoir, it is difficult to use a conventional oil displacement agent to achieve effective results. Therefore, it is necessary to find a polymer complex that can effectively inhibit the conductivity of macropore and fracture systems^55^. If this kind of polymer compound is used in the process of gas displacement, it can restrict the flow in the dominant channel and effectively prevent the one-way inrush of CO_2_, thus greatly improving the displacement recovery.

**Figure 21:**
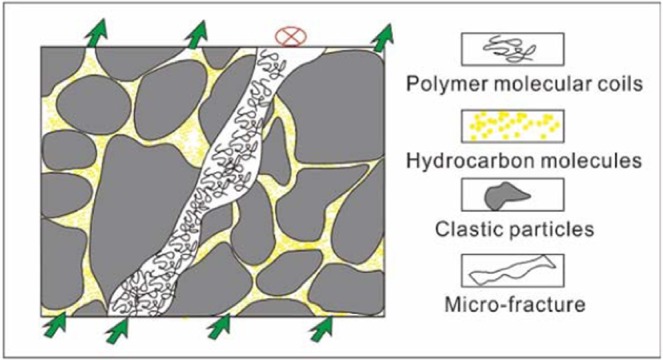
The mechanism of the oil displacement effect on the fracture reservoir after polymer injection.

At present, an injection water shutoff agent is the main method to deal with large pores and fractures during oilfield development. There are two main types of water shutoff agents, granular and polymer, and for a CO_2_ flooding reservoir, a cross-linked polymer water shutoff agent is the best choice^56^. When the water shutoff agent is injected into the underground reservoir, the polymer will be filled in the macropores and fractures. Under a high reservoir temperature, the hydroxymethyl group in the crosslinking agent and the amide group in the polymer will co-condensate; at the same time, the hydroxymethyl group in the polymer and the hydroxymethyl group of the crosslinking agent will self-condensate. Because of the different hydroxymethyl positions involved in the reaction, the polymer network structure can be formed. With the continuous cross-linking reaction, the volume of the complex molecular groups formed by the reaction will become increasingly larger and the viscosity of the injected liquid system will gradually increase, which will eventually plug the reservoir macropores or fractures (Fig. 21).

**Figure 22:**
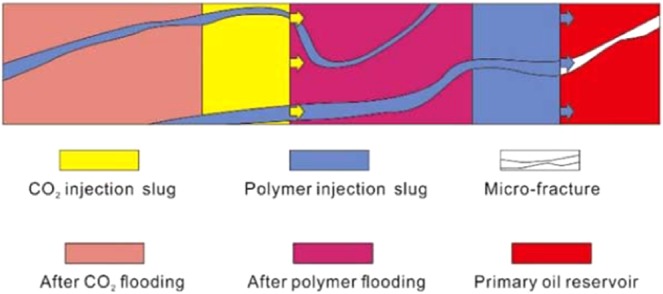
Principle of gas-liquid alternating injection flooding.

There are cracks in the Yanjia-Yongan sandy conglomerate reservoir, but the number of cracks is not excessive, and the distribution density is not very high. Therefore, it cannot be regarded as a fractured reservoir for special development. However, if CO_2_ flooding is used directly to inject in this reservoir, it is difficult to achieve long-term oil displacement effect due to the limitation of the reservoir properties. Our comprehensive analysis shows that the gas-liquid alternative injection (slug displacement) is more suitable for this reservoir, which can make full use of the advantages of carbon dioxide flooding and polymer flooding and thus offset the effect of reservoir fractures on oil displacement efficiency. First, a certain volume of liquid (water solution added with the polymer) is continuously injected into the formation. After a certain period, the polymer molecules in the liquid will fully occupy the macropore and fracture systems and reduce the heterogeneity of the reservoir. Then, CO_2_ is injected continuously for a certain period. At this time, CO_2_ will avoid blocked macropores and fractures and will turn to other homogeneous reservoirs, producing oil displacement. This process can effectively control the one-way gas burst and maximize the efficiency of the gas drive (Fig. 22).

**Figure 23:**
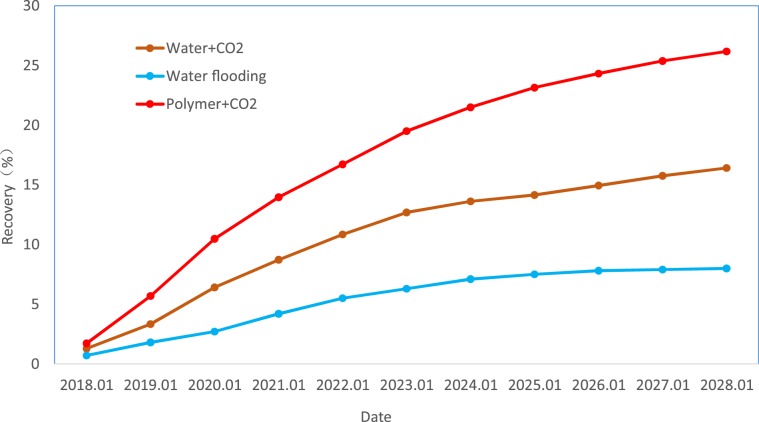
Contrast diagrams of displacement effect curves of different methods.

Figure 23 presents the oil displacement efficiency comparison curve of the Yanjia-Yongan glutenite reservoir using different displacement methods. The graph shows that the displacement method of alternating the injection of the gas (CO_2_) - liquid (polymer solution) can effectively inhibit the conductivity of reservoir fractures and macropores, increase the sweep area of the displacement agent, and improve oil displacement efficiency. After approximately 10 years of development, the total oil recovery of the reservoir can reach approximately 30%, while that of ordinary water flooding is less than 10%. This displacement method can greatly improve the oil recovery of the reservoir.

### Application prospects of carbon dioxide flooding in glutenite reservoirs

According to the actual data statistics, the total reserves of the discovered glutenite reservoirs in the Bohaiwan Basin have reached 700 million tons, accounting for approximately 8% of the total reserves in the basin. However, the conventional water flooding recovery of such reservoirs is relatively low; at present, the average is less than 10% due to low-efficiency development reservoirs. According to the research results of this paper, if the allocation relationship between reservoir parameters and injection fluids is properly handled, the glutenite reservoir can use CO_2_ for oil displacement under certain conditions, and the effect will be quite robust. If all the glutenite reservoirs in the basin can be successfully subjected to CO_2_ flooding, great economic benefits will result. Table 4 is the benefit prediction table of carbon dioxide flooding for glutenite reservoirs in the Bohaiwan Basin.

**Table 4:** Application prospects of CO_2_ flooding in glutenite reservoirs in the Bohaiwan Basin.

Reservoir of glutenite reservoir (10^8^ t)	Average recovery (%)	Replacement rate (t/t)	CO_2_ consumption (10^8^ t)
7.8	30	0.55	1.287

## Conclusion

1. In this paper, the glutenite reservoir is finely divided into different levels, and the sedimentary facies and sand body distribution of each level are predicted to determine a reasonable vertical development unit. On this basis, the physical properties and micro pore structure characteristics of glutenite reservoir are described and evaluated. The MMP of CO2 flooding in Glutenite is calculated, it is little difference between the current reservoir pressure and the calculated MMP. Therefore, miscible flooding technology can be reasonably developed in the study area.
2. The factors influencing CO_2_ flooding of the glutenite reservoir are analysed via reservoir numerical simulation technology. The effects of reservoir thickness, reservoir heterogeneity, macropores, dominant channels and fracturing on CO_2_ flooding efficiency are evaluated individually. The results show that: the thicker the reservoir is, the more likely the gas is to overlap by gravity, and the more unsuitable it is for CO_2_ flooding; The stronger the heterogeneity of the reservoir, the more uneven the spread range of CO_2_ flooding, and the lower the recovery efficiency. The large pores and dominant channels will increase the risk of one-way rapid gas breakthrough, which is not conducive to the improvement of CO_2_ flooding efficiency. Based on these research results, a set of reasonable parameter system is established in this paper, and the evaluation system of CO_2_ flooding effect in glutenite reservoir is established.
3. The initial evaluation results show that the conditions for CO_2_ flooding in the study area are not sufficient, because its thickness, heterogeneity and macropores are not suitable for the overall CO_2_ flooding. However, according to the geological characteristics of glutenite, a new CO_2_ flooding strategy has been designed in this study, and some adverse conditions have been improved to make the application of CO_2_ flooding more reasonable here. These improved techniques include: three-dimensional well pattern (upper and lower development well pattern is designed, it can solve the disadvantages caused by large reservoir thickness), reasonable injection mode (which can solve the adverse effects caused by heterogeneity), and testing the appropriate injection agent (which can solve the effects caused by fractures and large pores).

## Data Availability

The original data used in this paper are all from the real analysis, test, experiment and numerical simulation. The corresponding author can ensure the feasibility and authenticity of these data.

## References

[1] Zhao Y (2016). Visualization of asphaltene deposition effects on porosity and permeability during CO_2_ flooding in porous media. J. Journal of Visualization..

[2] Zhang Y (2015). CO_2_ foam flooding for improved oil recovery: Reservoir simulation models and influencing factors. J. Journal of Petroleum Science and Engineering..

[3] Lee S, Yun S, Kim J (2019). Development of novel sub-ambient membrane systems for energy-efficient post-combustion CO_2_ capture. J. Applied Energy..

[4] Connell L (2015). An investigation into the integrity of wellbore cement in CO_2_ storage wells: Core flooding experiments and simulations. J. International Journal of Greenhouse Gas Control..

[5] Xu C (2019). Insight into micro-mechanism of hydrate-based methane recovery and carbon dioxide capture from methane-carbon dioxide gas mixtures with thermal characterization. J. Applied Energy..

[6] Costa I (2019). Placing hubs in CO_2_ pipelines: An application to industrial CO_2_ emissions in the Iberian Peninsula. J.Applied Energy..

[7] Lan Y (2019). A review of microscopic seepage mechanism for shale gas extracted by supercritical CO_2_ flooding. J. Fule..

[8] Han J (2018). Effects of CO_2_ miscible flooding on oil recovery and the alteration of rock properties in a carbonate reservoir. J. Journal of CO2 Utilization..

[9] Zhai Z (2008). Pattern and potential of petroleum reserves growth in the Bohaiwan Basin. J. Oil and Gas Geology..

[10] Taşgin CK (2009). Analysis of soft-sediment deformation structures in Neogene fluvio-lacustrine deposits of Çaybağı Formation, Eastern Turkey. J. Sedimentary Geology..

[11] Cao Y (2014). The particle texture characteristics of sandy conglomerate in the nearshore subaqueous fan of upper ES_4_ in the Yanjia area, Dongying depression. J. Natural Gas Geoscience..

[12] Yan J (2011). Identification method of effective reservoir for glutenite body using well logging based on rock texture. J. Journal of Tongji University (natural science)..

[13] Qiu L (2018). Sedimentary characteristics and spatial distribution of coarse clastic rocks of fan delta in Changdi fault zone in Gubei sub-sag, Jiyang Depression. J. Oil and Gas Geology..

[14] Song M (2012). Fine division and correlation of conglomerate sedimentary cycles in Yanjia area of Dongying depression. J. Acta Petrolei Sinica..

[15] Mao Q (2010). Logging recognition of conglomerate in deep northern steep slope of Dongying depression. J. Petroleum Geology and Recovery Efficiency..

[16] Wang Q (2012). Classification of seismic sedimentary cycles of glutenite in the eastern Dongying Sag. J. Journal of Oil and Gas Technology..

[17] Zhao H (2010). The study on the methods for the depositional stage division of Glutenite body-a case of the actic region in the north of Dongying Depression. J. Petroleum Geophysics..

[18] Zhang H (2010). Fine stratigraphic classification and correlation for extremely thick glutenite in Yanjia oilfield. J. Oil. Geophysical Prospecting..

[19] Shu N (2016). Fracture prediction of sand-gravel body by multi-attribute fusion based on seismic imaging theory: A case study of the third member of Shahejie formation in northern zone of Chexi area in Jiyang depression. J. Petroleum Geology and Recovery Efficiency..

[20] Liu Z (2010). Application of glutenite reservoir predicting technique in the steep slope of Biyang depression. J. Geological Science and Technology Information..

[21] Wu Z (2016). Quantitative lithology identification technology of complex sand-conglomerate bodies. J. Lithologic Reservoirs..

[22] Zhu X (2013). On the differences of reservoir quality of shahejie the formation in steep slope zones of Jiyang sag. J. Acta Sedimentologica Sinica..

[23] Tian X (2014). Study on the heterogeneous characterization method of the internal sedimentary cycle in sand-gravel body reservoir. J. Journal of University of Science and Technology of China..

[24] Zhang L (2015). Types and distribution of diagenetic alterations in the nearshore subaqueous fan of the upper ES_4_ in Shengtuo area of Dongying depression. J. Natural Gas Geoscience..

[25] Yu H (2005). Water injection development test of Yanjia glutenite reservoir in Shengli oilfield. J. West-china Exploration Engineering..

[26] Yuan M (2002). Full scale developing thin glutinite extra-heavy oil in block Cao 13 by horizontal well thermal recovery. J. Special Oil and Gas Reservoirs..

[27] Cui C (2016). Inter well interference test design and conformation of inter well connectivity in Yong-1 glutenite reservoir. J. Journal of Liaoning Technical University (Natural Science)..

[28] Li C (2018). Gas channeling influencing factors and patterns of CO_2_ flooding in ultra-low permeability oil reservoir. J. Special Oil and Gas Reservoirs..

[29] Xu H (2010). Simulation experiment of oil displacement by CO_2_ injection into long core: an example from Fulin 3 member, Taixing oilfield. J. Natural Gas Exploration and Development..

[30] Gu L (2007). Experimental research of reservoir physical changes induced by CO_2_ flooding. J. Journal of oil and Gas Technology..

[31] Torabi F (2012). The evaluation of variable-injection rate water flooding, immiscible CO_2_ flooding, and water-alternating-CO_2_ injection for heavy oil recovery. J. Liquid Fuels. Technology..

[32] Bikkina P (2016). Influence of wettability and permeability heterogeneity on miscible CO_2_, flooding efficiency. J. Fuel..

[33] Alsumaiti AM (2018). Laboratory study of CO_2_ foam flooding in high temperature, high salinity carbonate reservoirs using Co-injection Technique. J. Energy & Fuels..

[34] Van SL (2018). Effective prediction and management of a CO_2_ flooding process for enhancing oil recovery using artificial neural networks. J. Journal of Energy Resources Technology..

[35] Wang J (2016). CO_2_ miscible flooding influence degree analysis of reservoir heterogeneity in low permeability reservoir. J. International. Journal of Oil Gas and Coal Technology..

[36] Moreno RZ (2011). Comparison of residual oil saturation for water and supercritical CO_2_ flooding in a long core, with live oil at reservoir conditions. J. Journal of Porous Media, v..

[37] Hoffman BT (2014). CO_2_ Flooding to increase recovery for unconventional liquids-rich reservoirs. J. Journal of Energy Resources Technology..

[38] Al-Bayati D (2018). An Experimental Investigation of Immiscible-CO_2_-Flooding Efficiency in Sandstone Reservoirs: Influence of Permeability Heterogeneity. J. SPE Reservoir Evaluation & Engineering..

[39] Ding M (2017). Oil recovery from a CO_2_ injection in heterogeneous reservoirs: The influence of permeability heterogeneity, CO_2_-oil miscibility and injection pattern. J. Journal of Natural Gas Science and Engineering..

[40] Al-Bayati D (2018). Influence of Permeability Heterogeneity on Miscible CO_2_ Flooding Efficiency in Sandstone Reservoirs: An Experimental Investigation. J. Transport in Porous Media..

[41] Al-Bayati D (2019). Insights into immiscible supercritical CO_2_ EOR: An XCT scanner assisted flow behaviour in layered sandstone porous media. J. Journal of CO2 Utilization..

[42] Al-Bayati D (2018). Insight investigation of miscible SCCO_2_ Water Alternating Gas (WAG) injection performance in heterogeneous sandstone reservoirs. J. Journal of CO2 Utilization..

[43] Li X (2016). Feasibility of produced gas reinjection during CO_2_ flooding and its influence on displacement efficiency. J. Petroleum Geology and Recovery Efficiency..

[44] Gao S (2013). Experimental study on anti-channeling during CO_2_ flooding for low permeability reservoirs. J. Special Oil and Gas Reservoirs..

[45] Wang G (2015). A new screening method of low permeability reservoirs suitable for CO_2_ flooding. J. Petroleum Exploration and Development..

[46] Zhang M, Guo P (2000). Theoretical research and application of the capillary pressure effect on the phase equilibrium in oil and gas system. J. Natural gas industry..

[47] Shao X (2018). Sedimentary facies boundary division of glutenite bodies in the upper ES4 of northern Minfeng zone in Dongying sag. J. Geological Science and Technology Information..

[48] Zhang S (2018). Prediction of sand-conglomerate reservoirs via seismic facies controlled inversion in the Lower ES_3_ of the northern steep slope of the Chexi sag. J. Earth Science Frontiers..

[49] Cao Y (2018). Characteristics of low-permeability clastic reservoirs and genesis of relatively higlrquality reservoirs in the continental rift lake basin: a case study of Paleogene in the Dongying sag, Jiyang depression. J. Acta Petrolei Sinica..

[50] Wang J (2014). Initial gas full-component simulation experiment of Ban-876 underground gas storage. J. Journal of Natural Gas Science and Engineering..

[51] Hu W (2018). Porous flow mechanisms and mass transfer characteristics of CO_2_ miscible flooding after water flooding. J. Acta Petrolei Sinica..

[52] Chen J (2018). Influence of pressure and CO_2_ content on the asphaltene precipitation and oil recovery during CO_2_ flooding. J. Petroleum Science & Technology..

[53] Xu B (2017). CO_2_ miscible flooding in low permeability sandstone reservoirs and its influence on crude oil properties. J. Petroleum Science & Technology..

[54] Amirian E (2018). Performance forecasting for polymer flooding in heavy oil reservoirs. J. Fuel..

[55] Riswati S (2018). Experimental analysis to design optimum phase type and salinity gradient of alkaline surfactant polymer flooding at low saline reservoir. J. Journal of Petroleum Science and Engineering..

[56] Zhao F (2007). Research advances of chemicals for oil production. J. Journal of China University of Petroleum..

[57] Yelling W, Metcalfe R (1980). Determination and Prediction of CO_2_ Minimum Miscibility Pressures. J. Journal of Petroleum Technology..

[58] Alston R, Kokolis G, James C (1985). CO_2_ Minimum Miscibility Pressure: A Correlation for Impure CO_2_ Streams and Live Oil Systems. J. SPE Journal..

[59] Orr F, Silva M (1987). Effect of Oil Composition on Minimum Miscibility Pressure-Part 2: Correlation. J. SPE Reservoir Engineering..

[60] Glaso O (1985). Generalized minimum miscibility pressure correlation. J. Society of Petroleum Engineers Journal..

